# Case Report: Temporal dissociation between peripheral B-cell pharmacodynamics and renal remission during sequential rituximab and telitacicept-containing therapy in childhood secondary steroid-resistant nephrotic syndrome

**DOI:** 10.3389/fimmu.2026.1958047

**Published:** 2026-10-20

**Authors:** Shaofan Hu, Qun Li, Haifeng Qi, Weimin Zheng, Xiaojie Peng, Rui Fu

**Affiliations:** Department of Nephrology, Jiangxi Provincial Children’s Hospital, Nanchang, China

**Keywords:** BAFF/APRIL, case report, rituximab, secondary steroid-resistant nephrotic syndrome (SRNS), telitacicept

## Abstract

Rituximab (RTX) can improve outcomes in children with frequently relapsing or steroid-dependent nephrotic syndrome, although a marked reduction in circulating B cells does not invariably coincide with renal remission. We report a girl who developed frequently relapsing/steroid-dependent nephrotic syndrome after an initial steroid-sensitive course, with kidney biopsy findings supporting minimal change disease. Following a relapse in 2024, persistent nephrotic-range proteinuria despite infection control and 4 weeks of standard-dose prednisone plus tacrolimus led to classification as secondary steroid-resistant nephrotic syndrome. She subsequently received two RTX infusions of 375 mg/m² (340 mg) 49 days apart. The last available pre-RTX peripheral CD19-positive B-cell count was 99.78 cells/μL and was 1.63 cells/μL 13 days after the first infusion; it had risen to 10.43 cells/μL immediately before the second infusion, and then decreased again to 1.29 cells/μL; however, proteinuria persisted. Telitacicept 80 mg once weekly was added to prednisone and tacrolimus on December 11, 2024. Peripheral B-cell reconstitution had begun before complete remission, and urine protein became negative 91 days after telitacicept initiation and 132 days after the second RTX infusion. Telitacicept was discontinued in May 2025 because of financial constraints, but remission was maintained. A transient increase in proteinuria during a respiratory tract infection subsequently resolved without restarting telitacicept. No serious treatment-related reactions were observed. This case illustrates a temporal dissociation between measured peripheral CD19-positive B-cell pharmacodynamics after RTX and complete renal remission. Remission was documented later during telitacicept-containing therapy; however, the prolonged RTX dosing interval, uncertain early RTX exposure, continued prednisone and tacrolimus, and natural disease fluctuations preclude causal attribution to telitacicept and do not establish biological RTX resistance. These observations support prospective evaluation of peripheral and tissue B-cell kinetics, RTX pharmacokinetics, and sequential CD20 and BAFF/APRIL targeting.

## Introduction

Childhood idiopathic nephrotic syndrome is characterized by substantial clinical and immunological heterogeneity. Most children are glucocorticoid-sensitive at initial presentation, but some subsequently develop frequently relapsing or steroid-dependent nephrotic syndrome (FRNS/SDNS). A small proportion of children fail to achieve complete remission after 4 weeks of standard-dose daily prednisone or prednisolone during a subsequent relapse and consequently develop secondary or late-onset steroid-resistant nephrotic syndrome (SRNS) (1, 2).

By targeting CD20, rituximab (RTX) eliminates most circulating mature B cells and can prolong relapse-free survival and reduce glucocorticoid exposure in children with complicated FRNS/SDNS (3). However, the magnitude and duration of peripheral B-cell reduction are pharmacodynamic observations and are not equivalent to renal remission. Memory B-cell reconstitution, anti-RTX antibodies, and inadequate drug exposure may all contribute to heterogeneity in treatment response (4–7). In immune thrombocytopenia, RTX-resistant splenic memory B cells with reduced surface CD20 and BCR-associated molecules have been identified, with epitope masking and CD20 internalization among the proposed explanations for loss of detectable surface CD20 (8). Although these findings cannot be directly extrapolated to nephrotic syndrome, they underscore that peripheral blood counts may not fully characterize the residual B-cell compartment. The identification of anti-nephrin autoantibodies and interferon-related immune networks in childhood nephrotic syndrome further suggests that some podocytopathies may have humoral immune features (9, 10).

B-cell activating factor (BAFF) and a proliferation-inducing ligand (APRIL) contribute to B-cell maturation and survival and support signaling in plasmablasts and plasma cells. Abnormal expression of BAFF, APRIL, and their receptors has been reported in childhood nephrotic syndrome (11, 12). Telitacicept is a transmembrane activator and calcium-modulator and cyclophilin ligand interactor-Fc (TACI-Fc) fusion protein that neutralizes both BAFF and APRIL (13). Renal evidence remains limited, with only isolated reports in minimal change disease and membranous nephropathy, together with emerging experience using telitacicept sequentially after RTX in refractory immune-mediated glomerular disease (14–16). We report a child with secondary SRNS who developed a marked reduction in measured peripheral CD19-positive B cells after RTX without concurrent renal remission and subsequently had remission documented during telitacicept-containing therapy. This case highlights the dissociation between peripheral pharmacodynamic and renal clinical responses and provides a hypothesis-generating rationale for prospective study of B-cell-targeted treatment sequencing.

## Case report

### Clinical course and diagnostic assessment

A girl first presented in July 2017 at 4 years of age with edema, oliguria, hypoalbuminemia, and heavy proteinuria. Urinary protein became negative after 1 week of full-dose prednisone, consistent with steroid-sensitive nephrotic syndrome. From 2018 to 2023, she experienced approximately four to five relapses per year during infections or glucocorticoid tapering. Most relapses responded again to treatment, but remission was short-lived, fulfilling the criteria for FRNS/SDNS. She received tacrolimus from February 2018 to May 2020, during which urinary protein remained negative for a maximum of approximately six months. From November 2020 to November 2021, treatment was switched to mycophenolate mofetil (MMF), with the longest remission lasting approximately 2 months. Because its efficacy was not superior to that of tacrolimus, tacrolimus was subsequently resumed. There was no known family history of nephrotic syndrome or chronic kidney disease. Because of financial constraints and concerns about potential drug-related adverse effects, the family declined RTX and cyclophosphamide, and they also declined genetic testing. The overall clinical course is summarized in **Table 1**.

**Table 1:** Key clinical timeline.

Time	Key event/intervention	Outcome and key data
July 2017 (age 4 years)	Initial nephrotic syndrome; full-dose prednisone	Proteinuria became negative within 1 week; SSNS was diagnosed.
2018–2023	Recurrent relapses and steroid-sparing immunosuppression	Approximately 4–5 relapses per year; FRNS/SDNS was established.
May 27, 2021	Kidney biopsy	Findings supported MCD; unsampled early FSGS could not be excluded.
August 16–September 12, 2024	Relapse treated with prednisone 50 mg/day and TAC 1 mg every 12 hours	Twenty-four-hour urinary protein decreased from 5.37 to 2.91 g/day but persisted after 4 weeks; secondary SRNS was diagnosed.
September 12, 2024	First RTX infusion, 340 mg	The last available pre-RTX CD19-positive B-cell count was 99.78 cells/μL; it was 1.63 cells/μL by September 25; complete remission was not achieved.
October 31, 2024	Second RTX infusion, 340 mg	The CD19-positive B-cell count was 10.43 cells/μL and 24-hour urinary protein was 2.19 g/day.
December 1–11, 2024	Post-RTX assessment; telitacicept 80 mg once weekly initiated	The CD19-positive B-cell count was 1.29 cells/μL; urinary protein was 1.66-2.00 g/day. Serum RTX was 0.01 μg/mL and anti-RTX antibodies were negative.
February 1–March 12, 2025	Telitacicept and background therapy continued	The CD19-positive B-cell count increased to 32.85 cells/μL before remission; urinary protein became negative and decreased to 0.10 g/day.
May 16, 2025	Telitacicept discontinued because of financial pressure	Remission was maintained after discontinuation.
September 28–October 30, 2025	Infection-associated transient proteinuria and final follow-up	24-hour urinary protein decreased from 2.21 g/day on September 28 to 0.25 g/day on October 1 and 0.10 g/day at the final follow-up, without restarting telitacicept.

FRNS, frequently relapsing nephrotic syndrome; FSGS, focal segmental glomerulosclerosis; MCD, minimal change disease; RTX, rituximab; SDNS, steroid-dependent nephrotic syndrome; SRNS, steroid-resistant nephrotic syndrome; SSNS, steroid-sensitive nephrotic syndrome; TAC, tacrolimus.

Kidney biopsy was performed on May 27, 2021, and yielded two tissue cores. Light microscopy showed 39 glomeruli without global or segmental sclerosis. Only one glomerulus was available for immunofluorescence, which showed IgA (±), IgM (+), and IgG (±), with negative staining for C1q, C3, and C4. Electron microscopy demonstrated diffuse foot-process effacement and microvillous transformation without electron-dense deposits. These findings supported minimal change disease, although unsampled early focal segmental glomerulosclerosis could not be excluded.

In August 2024, she was admitted with a relapse after a gastrointestinal infection. At admission, her blood pressure was 101/73 mmHg, height was 118 cm, and body weight was 28 kg. She had marked generalized edema. Serum albumin was 6.33 g/L, 24-hour urinary protein excretion was 5.37 g/day, serum creatinine was 35.91 μmol/L, and immunoglobulin G (IgG) was 1.67 g/L. Heavy proteinuria continued after the infection was controlled. Prednisone was increased to 50 mg/day (approximately 2 mg/kg/day based on a post-diuresis weight of 25 kg), and tacrolimus was given at 1 mg every 12 hours; the trough concentration was 6.67 ng/mL. After completion of 4 weeks of standard-dose treatment, 24-hour urinary protein excretion remained approximately 2.91 g/day. Given her clearly documented history of steroid sensitivity, the condition was classified as secondary SRNS (1, 2). Methylprednisolone pulse therapy and cyclophosphamide were not selected because of the risk of infection, cumulative immunosuppressive exposure, and the family’s preferences. In view of her previous complicated FRNS/SDNS, persistent disease activity, and cumulative treatment burden, RTX therapy was initiated.

### RTX treatment and pharmacodynamic assessment

Screening for hepatitis B, hepatitis C, human immunodeficiency virus, tuberculosis, Epstein–Barr virus, and cytomegalovirus before RTX treatment revealed no abnormalities. With intravenous immunoglobulin support, the patient received intravenous RTX at 340 mg, equivalent to 375 mg/m², on September 12 and October 31, 2024, and was given trimethoprim–sulfamethoxazole for infection prophylaxis. The two RTX infusions were 49 days apart. The last available pre-RTX CD19-positive B-cell count was 99.78 cells/μL; the count was 1.63 cells/μL 13 days after the first infusion. The count had risen to 10.43 cells/μL immediately before the second infusion on day 49 and subsequently decreased again to 1.29 cells/μL 31 days after the second infusion. Thus, RTX produced a marked peripheral CD19-positive B-cell reduction, but the effect was not continuously sustained between the two infusions. This pharmacodynamic change was not accompanied by contemporaneous complete renal remission. Twenty-four-hour urinary protein varied from 1.66 to 4.36 g/day, and complete remission was not reached.

At 37 days following the second RTX infusion, the serum RTX concentration was 0.01 μg/mL and the anti-RTX antibody concentration was 0.02 ng/mL, below the 2 ng/mL positivity threshold. This single late measurement showed only that little circulating RTX remained at that time and provided no evidence of anti-drug immunogenicity. It could not establish earlier systemic exposure or the duration of clinically relevant drug levels. Because early serial serum concentrations and urinary RTX measurements were unavailable, urinary loss or accelerated clearance during heavy proteinuria could not be excluded.

### BAFF/APRIL blockade, follow-up, and safety

Proteinuria remained despite two RTX infusions and a marked, but incompletely sustained, peripheral CD19-positive B-cell reduction, and the family did not wish to pursue another RTX dose. After discussion, telitacicept 80 mg was started subcutaneously once weekly on December 11, 2024, while prednisone 50 mg/day and tacrolimus 1 mg every 12 hours were continued. The dose was chosen with reference to the patient’s age, body weight, and available pediatric experience, and written consent was obtained for off-label use. At treatment initiation, urine protein was 2+ and 24-hour urinary protein excretion was 2.00 g/day. Proteinuria fluctuated during the first weeks. By February 1, the CD19-positive B-cell count had risen to 32.85 cells/μL, before remission was documented. On March 12, urinalysis first became negative for protein and 24-hour urinary protein was 0.10 g/day. This occurred approximately 132 days after the second RTX infusion and 91 days after telitacicept initiation. Morning urinalysis and urine dipstick assessments remained negative for protein during subsequent consecutive follow-up visits. Serial 24-hour urinary protein measurements are presented in **Supplementary Table S1**. Prednisone was gradually tapered, and the tacrolimus dose was not increased, as shown in **Figure 1**.

**Figure 1:**
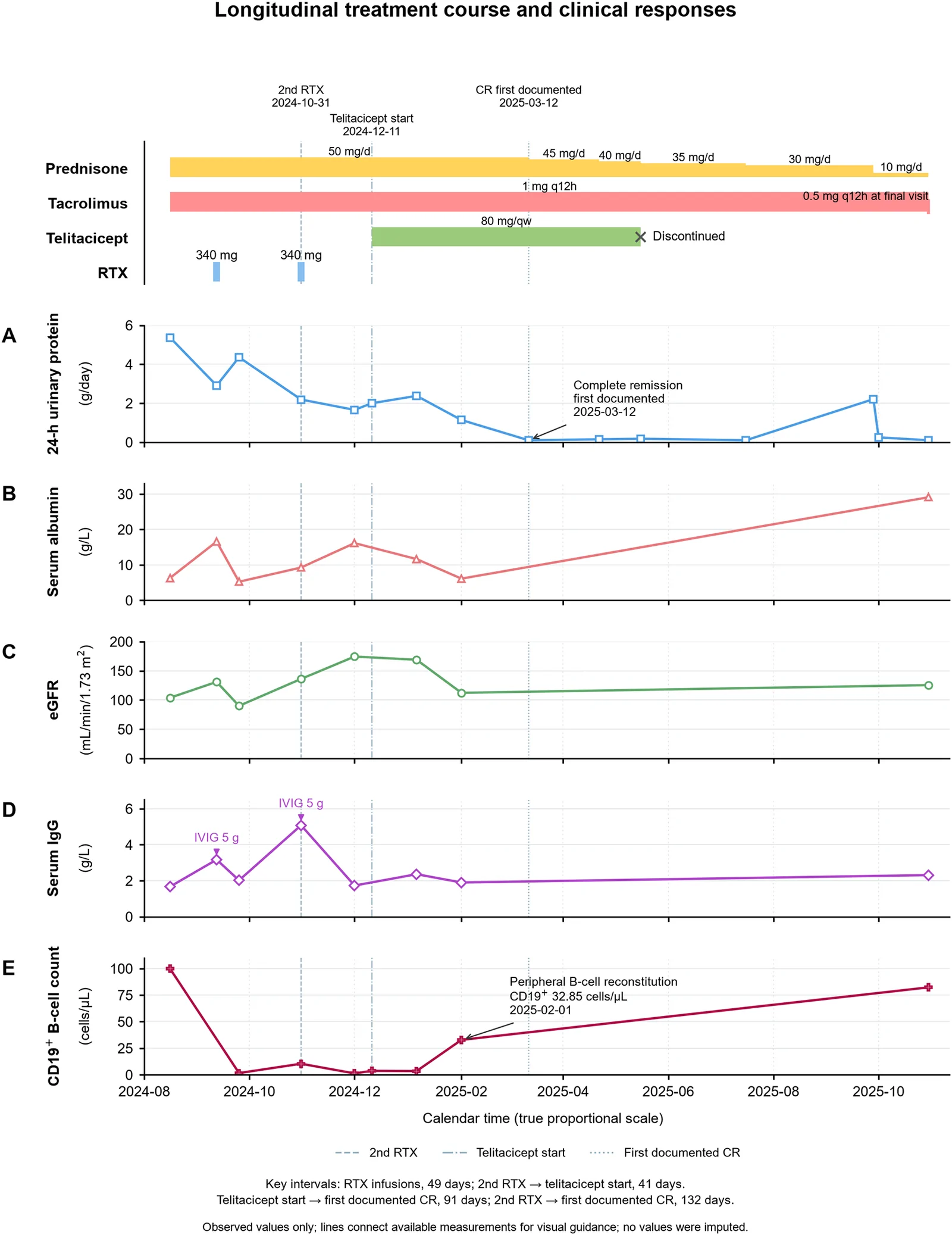
Longitudinal treatment course and clinical responses during sequential rituximab and telitacicept therapy. All treatment and laboratory data are shown on a shared true calendar-proportional x-axis. Five aligned panels show 24-hour urinary protein **(A)**, serum albumin **(B)**, eGFR **(C)**, serum IgG **(D)**, and peripheral CD19-positive B-cell count **(E)**, each with its own y-axis. Prednisone and tacrolimus regimens, telitacicept treatment (80 mg once weekly), and RTX infusions (340 mg each) are shown above the laboratory panels. The two RTX infusions were administered 49 days apart. Telitacicept was initiated 41 days after the second RTX infusion. Peripheral B-cell reconstitution was evident before complete remission, with the CD19-positive B-cell count reaching 32.85 cells/μL on February 1, 2025. Complete remission was first documented on March 12, 2025, 91 days after telitacicept initiation and 132 days after the second RTX infusion. Telitacicept was discontinued on May 16, 2025. IVIG 5-g annotations indicate IVIG administration. CR, complete remission; eGFR, estimated glomerular filtration rate; IgG, immunoglobulin G; IVIG, intravenous immunoglobulin; RTX, rituximab.

Beginning on May 16, 2025, the patient discontinued telitacicept on her own because of financial pressure. Urinalysis remained negative for protein after telitacicept discontinuation. During a respiratory tract infection on September 28, 2025, urinary protein increased transiently to 2+ on urinalysis and 24-hour urinary protein rose to 2.21 g/day. After 3 days of oral anti-infective treatment, urinalysis became negative for protein again on October 1, and 24-hour urinary protein decreased to 0.25 g/day without reinitiation of telitacicept. At the end of follow-up on October 30, 2025, urinary protein remained negative while the patient was receiving prednisone at 10 mg/day and tacrolimus at 0.5 mg every 12 hours. No serious infusion reactions, definite injection-site reactions, or allergic reactions occurred during RTX or telitacicept treatment. IgG was low at several points during active disease and B-cell-targeted treatment; however, some measurements followed intravenous immunoglobulin administration and were therefore difficult to interpret. Given the concurrent heavy proteinuria, hypoalbuminemia, and use of several immunosuppressive agents, neither the low IgG levels nor the infection risk could be assigned to a single drug. At the last follow-up, kidney function remained preserved, with a serum creatinine level of 31.00 μmol/L and an estimated glomerular filtration rate (eGFR) of 125.57 mL/min/1.73 m², calculated using the creatinine-based CKiD U25 equation. Continued monitoring was planned for proteinuria, kidney function, immunoglobulin levels, B-cell reconstitution, infections, and treatment-related adverse events.

## Discussion

The main value of this case lies in the longitudinal observation that a marked but incompletely sustained peripheral CD19-positive B-cell reduction after RTX did not coincide with complete renal remission. The measured circulating B-cell count fell markedly but was not continuously suppressed across the 49-day interval between infusions; proteinuria persisted, and remission was documented later during telitacicept-containing therapy after B-cell reconstitution had already begun. These observations underscore that total peripheral CD19-positive B-cell counts are pharmacodynamic markers and may not track the timing of renal response or fully characterize residual B-cell compartments.

This patient did not have initial-onset SRNS. She responded quickly to glucocorticoids at presentation and then had several years of FRNS/SDNS. The 2021 biopsy was interpreted as minimal change disease. This history supports a clinically suspected immune-mediated and potentially reversible component, but it does not establish the mechanism of the later steroid-resistant phase. RTX can induce complete or partial remission in some children with SRNS, although responses are variable (17). Important uncertainties remain. Genetic testing was not performed, and the biopsy was obtained more than three years before secondary steroid resistance developed; it may therefore not have reflected the later pathological phenotype. Unsampled or subsequently evolved focal segmental glomerulosclerosis and an underlying genetic podocytopathy remain possible. Accordingly, the later podocytopathy cannot be assumed to have been purely immune-mediated.

RTX targets CD20, whereas this case was monitored with total peripheral CD19-positive B-cell counts. These measurements do not resolve memory B-cell subsets and do not capture tissue-resident B cells or CD20-low or CD20-negative antibody-secreting cells. In pediatric nephrotic syndrome, memory B-cell reappearance may track disease activity more closely than recovery of the total CD19-positive B-cell population (4, 5). In immune thrombocytopenia, Crickx et al. identified RTX-resistant splenic memory B cells with undetectable surface CD20 and reduced expression of several BCR-associated molecules, including CD19, CD21, CD22, and CD79b; epitope masking and CD20 internalization were among the proposed explanations for loss of detectable surface CD20 (8). These data were obtained outside nephrotic syndrome and do not establish the same mechanism in this patient, but they further caution against equating a low circulating CD19-positive count with complete elimination of potentially pathogenic B-cell populations. The discovery of anti-nephrin autoantibodies and interferon-related immune activation also highlights the biological diversity of clinically similar podocytopathies (9, 10). Persistent proteinuria after marked peripheral CD19-positive B-cell reduction could therefore reflect CD20-low or CD20-negative antibody-secreting cells, residual immune cells in tissue, or podocyte injury that is not mainly B-cell dependent. None of these possibilities was assessed here. The course is better described as a separation between pharmacodynamic and clinical responses than as proven biological resistance to RTX.

RTX exposure is another important uncertainty. Each infusion used the standard dose of 375 mg/m², but the two infusions were administered 49 days apart. This differed from commonly used short-interval regimens: Iijima et al. administered 375 mg/m² once weekly for four doses in complicated FRNS/SDNS (3), and KDIGO 2025 notes that, in SRNS with nephrotic-range proteinuria, two 375 mg/m² infusions on days 1 and 8 may be preferable to achieve complete B-cell depletion (2). In this patient, the CD19-positive B-cell count rose from 1.63 cells/μL after the first infusion to 10.43 cells/μL immediately before the second, indicating that the initial reduction was not sustained across the full dosing interval. The prolonged interval may therefore have limited the persistence of the peripheral B-cell reduction, although each individual dose was standard. Thirty-seven days after the second infusion, serum RTX was 0.01 μg/mL, while anti-RTX antibodies remained below the positive threshold (6). This single late measurement cannot define earlier systemic exposure or the duration of clinically relevant drug levels. Active nephrotic syndrome may increase urinary loss and clearance of macromolecular drugs and shorten RTX exposure (7). Early serial serum concentrations and urinary RTX measurements were not available. The low late concentration could therefore represent either expected time-dependent decline or underexposure related to severe proteinuria. Accordingly, the absence of contemporaneous renal remission should not be interpreted as proven biological RTX resistance.

The BAFF/APRIL pathway provides a biological rationale for considering sequential B-cell-targeted treatment. BAFF/APRIL signaling has been implicated in childhood nephrotic syndrome, and telitacicept neutralizes both ligands (11–13). In this patient, peripheral B-cell reconstitution preceded remission, but serial BAFF/APRIL measurements, B-cell subset phenotyping, plasmablast/plasma-cell analyses, tissue-resident B-cell assessment, anti-nephrin antibody testing, and telitacicept pharmacokinetic/pharmacodynamic measurements were not performed. Therefore, the temporal sequence cannot demonstrate mechanistic complementarity between CD20 and BAFF/APRIL targeting. Reports of telitacicept-containing regimens in minimal change disease, membranous nephropathy, lupus nephritis, IgA nephropathy, and IgA vasculitis nephritis provide only indirect clinical rationale (14–16, 18, 19); differences in age, disease mechanism, and study design preclude direct extrapolation to childhood secondary SRNS. These observations should therefore be considered hypothesis-generating and a rationale for prospective mechanistic studies.

Several factors limit causal interpretation. Telitacicept was started 41 days after the second RTX infusion, and remission was first documented 91 days later, or 132 days after the second RTX infusion, while prednisone and tacrolimus were continued. A delayed RTX response, ongoing background immunosuppression, infection control, and spontaneous disease fluctuation could all have contributed. Remission persisted after telitacicept was stopped, and a later infection-related rise in proteinuria resolved without re-treatment. Thus, no dechallenge-rechallenge pattern was observed, and remission should be described as occurring during telitacicept-containing therapy rather than as a telitacicept-specific response. Other limitations include the absence of genetic testing and repeat biopsy, inconsistent methods of proteinuria assessment at some time points, a single late RTX concentration, and the absence of the mechanistic and pharmacokinetic measurements noted above. Interpretation of low IgG was also complicated by urinary loss, intravenous immunoglobulin, prior RTX exposure, and concomitant immunosuppression. The absence of serious adverse events does not establish long-term pediatric safety. Even with these limitations, the longitudinal data support proteinuria, rather than total peripheral CD19-positive B-cell counts, as the principal renal endpoint and justify prospective evaluation of B-cell-targeted treatment sequencing in patients characterized by contemporaneous genetic and pathological data.

## Conclusion

This case documents a temporal dissociation between a marked reduction in the measured peripheral CD19-positive B-cell count after RTX and complete renal remission in a child with secondary SRNS. The B-cell reduction was not continuously sustained between RTX infusions, and remission was documented later during telitacicept-containing therapy after peripheral B-cell reconstitution had begun. Thus, total circulating CD19-positive B-cell counts should be interpreted as pharmacodynamic markers rather than surrogate renal endpoints. The clinical course provides a hypothesis-generating rationale to prospectively study sequential CD20 and BAFF/APRIL targeting, but it does not demonstrate therapeutic complementarity. Given the 49-day RTX dosing interval, unmeasured early RTX exposure, continued prednisone and tacrolimus, and diagnostic uncertainty of the later podocytopathy, neither biological RTX resistance nor a telitacicept-specific remission effect can be inferred. Prospective studies incorporating contemporaneous pathology, genetic testing, pharmacokinetics, and detailed immune phenotyping are needed.

## Patient and caregiver perspective

The patient and her caregiver described repeated relapses, frequent hospital visits, and prolonged glucocorticoid and immunosuppressive therapy as major practical, psychological, educational, and financial burdens. The patient was particularly worried about future relapses and felt that the disease had disrupted her daily life and schooling. Both were encouraged when urinary protein became negative and hoped that remission could be maintained. However, the cost of weekly telitacicept injections was difficult to sustain, leading the family to discontinue treatment after remission was achieved. They hoped that future therapy would remain safe and effective while becoming less burdensome and more affordable.

## Data Availability

The original contributions presented in the study are included in the article/**Supplementary Material**, further inquiries can be directed to the corresponding author/s.

## References

[1] Trautmann A Boyer O Hodson E Bagga A Gipson DS Samuel S . IPNA clinical practice recommendations for the diagnosis and management of children with steroid-sensitive nephrotic syndrome. Pediatr Nephrol. (2023) 38:877–919. doi: 10.1007/s00467-022-05739-3 36269406 PMC9589698

[2] Kidney Disease: Improving Global Outcomes (KDIGO) Nephrotic Syndrome in Children Work Group . KDIGO 2025 clinical practice guideline for the management of nephrotic syndrome in children. Kidney Int. (2025) 107:S241–89. doi: 10.1016/j.kint.2024.11.007 40254391

[3] Iijima K Sako M Nozu K Mori R Tuchida N Kamei K . Rituximab for childhood-onset, complicated, frequently relapsing nephrotic syndrome or steroid-dependent nephrotic syndrome: a multicentre, double-blind, randomised, placebo-controlled trial. Lancet. (2014) 384:1273–81. doi: 10.1016/S0140-6736(14)60541-9 24965823

[4] Colucci M Carsetti R Cascioli S Casiraghi F Perna A Ravà L . B cell reconstitution after rituximab treatment in idiopathic nephrotic syndrome. J Am Soc Nephrol. (2016) 27:1811–22. doi: 10.1681/ASN.2015050523 26567244 PMC4884116

[5] Colucci M Angeletti A Zotta F Carsetti R Lugani F Ravà L . Age and memory B cells at baseline are associated with risk of relapse and memory B-cell reappearance following anti-CD20 treatment in pediatric frequently relapsing/steroid-dependent nephrotic syndrome. Kidney Int. (2023) 104:577–86. doi: 10.1016/j.kint.2023.06.013 37385541

[6] Bertrand Q Mignot S Kwon T Couderc A Maisin A Cambier A . Anti-rituximab antibodies in pediatric steroid-dependent nephrotic syndrome. Pediatr Nephrol. (2022) 37:357–65. doi: 10.1007/s00467-021-05069-w 34132894

[7] Counsilman CE Jol-van der Zijde CM Stevens J Cransberg K Bredius RGM Sukhai RN . Pharmacokinetics of rituximab in a pediatric patient with therapy-resistant nephrotic syndrome. Pediatr Nephrol. (2015) 30:1367–70. doi: 10.1007/s00467-015-3120-8 26054711 PMC4483248

[8] Crickx E Chappert P Sokal A Weller S Azzaoui I Vandenberghe A . Rituximab-resistant splenic memory B cells and newly engaged naive B cells fuel relapses in patients with immune thrombocytopenia. Sci Transl Med. (2021) 13:eabc3961. doi: 10.1126/scitranslmed.abc3961 33853929 PMC7610758

[9] Hengel FE Dehde S Lassé M Zahner G Seifert L Schnarre A . Autoantibodies targeting nephrin in podocytopathies. N Engl J Med. (2024) 391:422–33. doi: 10.1056/NEJMoa2314471 38804512

[10] Li QY Liu F Li X Kang M Bai L Tong T . Single cell analysis identified IFN signaling activation contributes to the pathogenesis of pediatric steroid-sensitive nephrotic syndrome. Biomark Res. (2025) 13:77. doi: 10.1186/s40364-025-00790-2 40413560 PMC12103753

[11] Forero-Delgadillo J Ochoa V Restrepo JM Torres-Canchala L Nieto-Aristizábal I Ruiz-Ordoñez I . B-cell activating factor (BAFF) and its receptors' expression in pediatric nephrotic syndrome is associated with worse prognosis. PLoS One. (2022) 17:e0277800. doi: 10.1371/journal.pone.0277800 36399480 PMC9674176

[12] Ling C Chen Z Hua L Zhang H Wu D Xi Y . Serum levels of B-cell activating factor and a proliferation-inducing ligand in children with steroid-sensitive nephrotic syndrome. Nephron. (2025) 149:384–91. doi: 10.1159/000544761 40010307

[13] Shi F Xue R Zhou X Shen P Wang S Yang Y . Telitacicept as a BLyS/APRIL dual inhibitor for autoimmune disease. Immunopharmacol Immunotoxicol. (2021) 43:666–73. doi: 10.1080/08923973.2021.1973493 34519594

[14] Li S Ding L Yang YJ Yang XD . Telitacicept for minimal change disease. Kaohsiung J Med Sci. (2023) 39:748–9. doi: 10.1002/kjm2.12719 37440467 PMC11895963

[15] Zhang L Jin H Wang D Wang Y . Case report: Successful treatment of refractory membranous nephropathy with telitacicept. Front Immunol. (2023) 14:1268929. doi: 10.3389/fimmu.2023.1268929 37915584 PMC10616774

[16] Chen Y Lei X Xu J Chen X Pan H Zhang Q . Belimumab versus telitacicept in sequential treatment after rituximab for refractory lupus nephritis: a real-world multicentre study. Lupus Sci Med. (2025) 12:e001296. doi: 10.1136/lupus-2024-001296 39762087 PMC11751942

[17] Chan EYH Sinha A Yu ELM Akhtar N Angeletti A Bagga A . An international, multi-center study evaluated rituximab therapy in childhood steroid-resistant nephrotic syndrome. Kidney Int. (2024) 106:1146–57. doi: 10.1016/j.kint.2024.09.011 39395629

[18] Lv J Liu L Hao C Li G Fu P Xing G . Randomized phase 2 trial of telitacicept in patients with IgA nephropathy with persistent proteinuria. Kidney Int Rep. (2023) 8:499–506. doi: 10.1016/j.ekir.2022.12.014 36938094 PMC10014376

[19] Li SX Hu RK Kong DL Xu J Bian Q Guo ZY . Efficacy of telitacicept in treating IgA vasculitis nephritis: a two-case report. Front Immunol. (2025) 16:1564242. doi: 10.3389/fimmu.2025.1564242 40375991 PMC12078309

