# Supplementary material for: Case Report: Temporal dissociation between peripheral B-cell pharmacodynamics and renal remission during sequential rituximab and telitacicept-containing therapy in childhood secondary steroid-resistant nephrotic syndrome
Source: Front Immunol. 2026 Oct 20;17:1958047. doi: 10.3389/fimmu.2026.1958047 (PMC13727527; doi:10.3389/fimmu.2026.1958047)
Supplement: Supplementary file 1 [file Table1.docx]

**Supplementary Table S1.** Longitudinal laboratory findings and therapeutic interventions before, during, and after sequential B-cell-targeted therapy.

| **Date** | **Laboratory findings** | | | | | | | **Therapeutic interventions** | | | | |
| --- | --- | --- | --- | --- | --- | --- | --- | --- | --- | --- | --- | --- |
|  | **Urine dipstick** **protein** | **24-h urinary** **protein (g/day)** | **Serum albumin** **(g/L)** | **IgG** **(g/L)** | **Serum creatinine** **(μmol/L)** | **eGFR** **(mL/min/1.73 m²)** | **CD19^+^ B-cell count (cells/μL)** | **Prednisone** **(mg/day)** | **Tacrolimus** **(mg/q12h)** | **RTX** **(mg)** | **IVIG** **(g)** | **Telitacicept** **(mg/qw)** |
| 2024-08-16 | 3+ | 5.37 | 6.33 | 1.67 | 35.91 | 103.99 | 99.78 | 50 | 1 | — | — | — |
| 2024-09-12 | 3+ | 2.91 | 16.59 | 3.17 | 28.50 | 131.03 | NA | 50 | 1 | 340 | 5 | — |
| 2024-09-25 | 3+ | 4.36 | 5.26 | 2.02 | 41.47 | 90.05 | 1.63 | 50 | 1 | — | — | — |
| 2024-10-31 | 2+ | 2.19 | 9.27 | 5.08 | 27.43 | 136.14 | 10.43 | 50 | 1 | 340 | 5 | — |
| 2024-12-01 | 2+ | 1.66 | 16.16 | 1.73 | 21.60 | 174.35 | 1.29 | 50 | 1 | — | — | — |
| 2024-12-11 | 2+ | 2.00 | NA | NA | NA | NA | 3.73 | 50 | 1 | — | — | 80 |
| 2025-01-06 | 2+ | 2.38 | 11.65 | 2.36 | 22.29 | 168.96 | 3.51 | 50 | 1 | — | — | 80 |
| 2025-02-01 | 2+ | 1.14 | 6.14 | 1.90 | 33.80 | 112.36 | 32.85 | 50 | 1 | — | — | 80 |
| 2025-03-12 | Negative | 0.10 | NA | NA | NA | NA | NA | 45 | 1 | — | — | 80 |
| 2025-04-22 | Negative | 0.12 | NA | NA | NA | NA | NA | 40 | 1 | — | — | 80 |
| 2025-05-16 | Negative | 0.13 | NA | NA | NA | NA | NA | 35 | 1 | — | — | Discontinued |
| 2025-07-16 | Negative | 0.10 | NA | NA | NA | NA | NA | 30 | 1 | — | — | — |
| 2025-09-28 | 2+ | 2.21 | NA | NA | NA | NA | NA | 10 | 1 | — | — | — |
| 2025-10-01 | Negative | 0.25 | NA | NA | NA | NA | NA | 10 | 1 | — | — | — |
| 2025-10-30 | Negative | 0.10 | 29.11 | 2.31 | 31.00 | 125.57 | 82.28 | 10 | 0.5 | — | — | — |

**Note:** In the medication columns, prednisone, tacrolimus, and telitacicept entries indicate the treatment regimen in use at each time point, whereas RTX and IVIG entries indicate doses administered on the corresponding date. An em dash (—) indicates no ongoing treatment or no administration, as applicable. “Discontinued” marks the date of treatment cessation. For laboratory variables, NA indicates that the measurement was not available or was not performed. eGFR was calculated using the age- and sex-specific creatinine-based CKiD U25 equation. eGFR, estimated glomerular filtration rate; IgG, immunoglobulin G; IVIG, intravenous immunoglobulin; RTX, rituximab.
